# Investigation of the Effect of Dispersion Medium Parameters on the Aggregative Stability of Selenium Nanoparticles Stabilized with Catamine AB

**DOI:** 10.3390/mi14020433

**Published:** 2023-02-11

**Authors:** Andrey V. Blinov, David G. Maglakelidze, Zafar A. Rekhman, Maria A. Yasnaya, Alexey A. Gvozdenko, Alexey B. Golik, Anastasiya A. Blinova, Maxim A. Kolodkin, Naiyf S. Alharbi, Shine Kadaikunnan, Muthu Thiruvengadam, Mohammad Ali Shariati, Andrey A. Nagdalian

**Affiliations:** 1 Department of Physics and Technology of Nanostructures and Materials, Physical and Technical Faculty, North Caucasus Federal University, 1 Pushkin Str., 355017 Stavropol, Russia; 2 Laboratory of Food and Industrial Biotechnology, Faculty of Food Engineering and Biotechnology, North Caucasus Federal University, 1 Pushkin Str., 355017 Stavropol, Russia; 3 Department of Botany and Microbiology, College of Science, King Saud University, Riyadh 11451, Saudi Arabia; 4 Department of Applied Bioscience, College of Life and Environmental Sciences, Konkuk University, Seoul 05029, Republic of Korea; 5 Department of Microbiology, Saveetha Dental College and Hospitals, Saveetha Institute of Medical and Technical Sciences, Saveetha University, Chennai 600077, Tamil Nadu, India; 6 Department of Scientific Research, K.G. Razumovsky Moscow State University of Technologies and Management (The First Cossack University), 73 Zemlyanoy Val, 109004 Moscow, Russia

**Keywords:** ζ-potential, transmission electron microscopy, average hydrodynamic radius, aggregative stability

## Abstract

This article presents the results of the synthesis of Se NPs stabilized by a quaternary ammonium compound—catamine AB. Se NPs were obtained by chemical reduction in an aqueous medium. In the first stage of this study, the method of synthesis of Se NPs was optimized by a multifactorial experiment. The radius of the obtained samples was studied by dynamic light scattering, and the electrokinetic potential was studied using acoustic and electroacoustic spectrometry. Subsequently, the samples were studied by transmission electron microscopy, and the analysis of the data showed that a bimodal distribution is observed in negatively charged particles, where one fraction is represented by spheres with a diameter of 45 nm, and the second by 1 to 10 nm. In turn, positive Se NPs have a diameter of about 70 nm. In the next stage, the influence of the active acidity of the medium on the stability of Se NPs was studied. An analysis of the obtained data showed that both sols of Se NPs exhibit aggregative stability in the pH range from 2 to 6, while an increase in pH to an alkaline medium is accompanied by a loss of particle stability. Next, we studied the effect of ionic strength on the aggregative stability of Se NPs sols. It was found that negatively charged ions have a significant effect on the particle size of the positive sol of Se NPs, while the particle size of the negative sol is affected by positively charged ions.

## 1. Introduction

Cationic surfactants are substances whose surface activity is caused by cations with long-chain hydrophobic radicals when dissolved in water. This class of substances is extremely promising for scientific research. They have been successfully used to stabilize various nanoscale materials, for example, Se NPs, silica nanospheres, and nanoscale ZnO [1,2,3,4]. They are one of the most effective stabilizers due to their adsorption ability on the surface of nanoparticles [5,6,7,8]. Scientists have found that cetyltrimethylammonium bromide (CTAB) can be adsorbed onto silver nanoparticles by strong chemisorption in an aqueous solution, which is accompanied by the stabilization of nanoparticles [9]. Alkyldimethylbenzylammonium chloride or catamine AB is a promising cationic surfactant. A lot of work aims to investigate the medicinal properties of catamine AB. Thus, in the work [10], the efficacy of the immobilized form of catamine AB in the treatment of purulent wounds of rats was investigated, which established a high rate of regeneration, and a positive effect on wound healing. Catamine AB is also used in wound treatment drugs with pronounced antimicrobial activity, having anti-allergic and anti-inflammatory effects, in eye drops having antiviral, antibacterial, and detoxifying effects [11,12,13]. Catamine AB is used as a stabilizer for Fe_2_O_3_ nanoparticles, gold nanorods, nanosilver with increased antibacterial activity, nulvalent iron nanoparticles used for targeted drug delivery, and silicon dioxide [14,15,16,17,18,19]. Electrochemical sensors that detect the antiviral drug tenofovir are being developed based on silver nanoparticles stabilized by catamine AB [20].

Due to the relevance of cationic surface substances and the broad prospects for the use of catamine AB, we conducted a study of the synthesis process of nanoscale selenium using a stabilizer—catamine AB.

Selenium is an important element for humans, bacteria, and animals. In the human body, the nutritional functions of selenium are achieved by 25 selenoproteins, they have antioxidant, anti-inflammatory, and antiviral properties [21]. Selenium is essential for the proper functioning of many aspects of the immune system. It affects both the innate and the acquired immune systems. Selenium-containing glutathione peroxidase protects neutrophils from oxygen-derived radicals [22]. Selenium deficiency, in turn, leads to mulberry heart disease, Kashin Beck’s disease, which is a bone and joint disease [23].

Se NPs, on the other hand, have significantly lower toxicity than their inorganic form [24,25]. Se NPs are a promising area of research due to their unique properties, such as high biological activity, bioavailability, and low toxicity [26,27]. Se NPs are widely used in medical diagnostics and drug delivery [28]. As Se NPs have a lower risk than inorganic selenium, they are used as a food additive, antioxidants, antimicrobial, and anticancer agents [29,30,31].

Due to the actual application of the material, the aim of the work was to develop and optimize the synthesis method and study Se NPs stabilized with alkyldimethylbenzylammonium chloride.

## 2. Materials and Methods

### 2.1. Synthesis of Se NPs Stabilized with Alkyldimethylbenzylammonium Chloride

**Figure 1:**
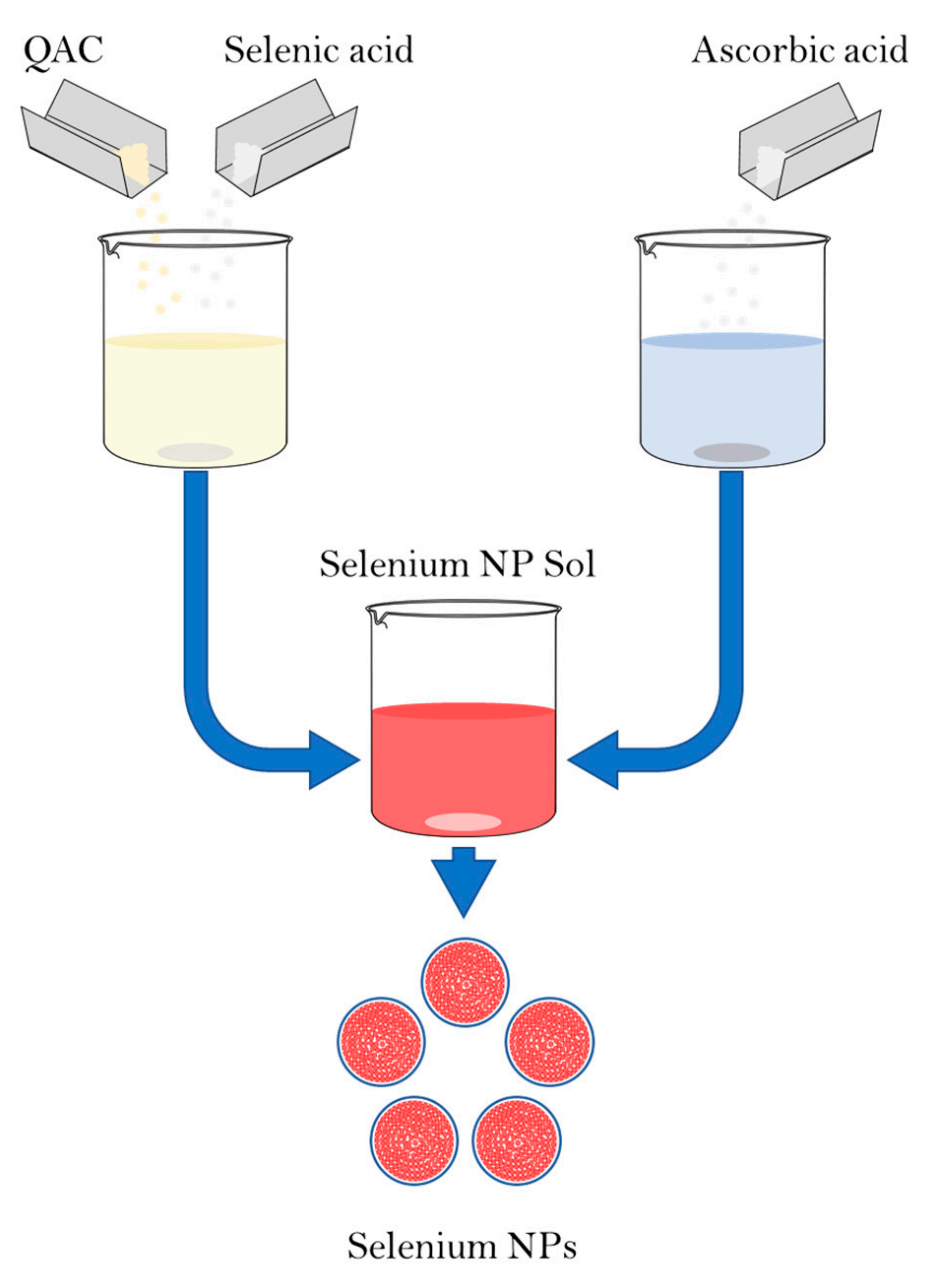
Scheme of synthesis of Se NPs stabilized with alkyldimethylbenzylammonium chloride (catamine AB).

The synthesis of Se NPs was carried out by a chemical reduction in aqueous medium in the presence of stabilizers. The synthesis scheme is shown in Figure 1. Alkyldimethylbenzylammonium chloride (catamine AB) (Vitareactive, Dzerzhinsk, Russia) was used as a stabilizer. Selenious acid was used as a selenium-containing precursor (INTRERHIM, St. Petersburg, Russia), and ascorbic acid was used as a reducing agent (Lenreactive, St. Petersburg, Russia).

The synthesis of Se NPs samples stabilized with alkyldimethylbenzylammonium chloride (catamine AB) was carried out in three stages. In the first stage, solutions were prepared with a different ratio between the amount of quaternary ammonium compounds and the amount of selenious acid. For this purpose, 0.036 M of the selenious acid solution was dissolved in 100 cm^3^ from 0.68 g to 5.24 g of quaternary ammonium compound, depending on the given ratio. In the second stage, 0.088 M ascorbic acid solution was prepared by dissolving 773.8 mg in 50 cm^3^ of distilled water. In the third stage, a solution of ascorbic acid was added dropwise to a solution of selenic acid and stabilizer under intensive stirring and the resulting sample was mixed for 5–10 min.

### 2.2. Methods of Investigation of Se NPs Stabilized with Alkyldimethylbenzylammonium Chloride (Catamine AB)

The microstructure of Se NPs samples was studied using a Carl Zeiss Libra 120 M transmission electron microscope (Carl Zeiss, Oberkochen, Germany). Nanoselenium samples were applied by ultrasonic dispersion of a solution of the test sample and water in a ratio of 1:1 on copper grids with a carbon substrate. No more than 3 days elapsed between the application of samples and scanning. The accelerating voltage amplitude of the Libra 120 M thermal emission gun (Carl Zeiss, Oberkochen, Germany) was 120 kV [32].

The size of Se NPs was studied by dynamic light scattering (DLS) on a Photocor-Complex device (Antek-97, Moscow, Russia). Computer processing of the obtained results was carried out using the DynaLS software (Antek-97, Moscow, Russia). For the research, samples of selenium nanoparticles were diluted four times with distilled water [33].

The ζ-potential was studied by acoustic and electroacoustic spectroscopy at the DT-1202 facility (Dispersion Technology, New York, NY, USA) [34].

Quantum chemical modeling of the process of stabilization of Se NPs with alkyldimethylbenzylammonium chloride was carried out in the QChem program using the IQmol molecular editor (Q-Chem, Pleasanton, CA, USA). The calculation was carried out on the equipment of the data processing center (Schneider Electric) of the North Caucasus Federal University. The calculation of the total energy and other characteristics was carried out with the following parameters: calculation: Energy; method: HF, basis: 3-21G, convergence—5, force field—Ghemical.

To study functional groups in the obtained samples, IR spectroscopy was used. IR spectra were recorded on an FSM-1201 IR-spectrometer with Fourier transform (Infraspek, Saints Petersburg, Russia). The measurement range was 500–4000 cm^− 1^.

### 2.3. Optimization of the Method of Synthesis of Se NPs Stabilized with Alkyldimethylbenzylammonium Chloride (Catamine AB)

To optimize the experimental parameters, a multifactorial experiment was performed with three input parameters and three levels of variation. Selenious acid, catamine AB, and ascorbic acid concentrations were selected as input parameters, the average hydrodynamic radius of particles and the ζ–potential were selected as output parameters. The levels of variation of variables are presented in Table 1.

Based on data of Table 1 we prepared an experiment matrix shown in Table 2.

### 2.4. Investigation of the Effect of the Active Acidity of the Medium on the Stability of Samples of Se NPs

To study the effect of the active acidity of the medium on the stability of the positive and negative sols of Se NPs, solutions with different active acidity of the medium were added to the samples in a ratio of 1:1. The solutions with different pH were obtained by the following method: first, a solution of a mixture of phosphoric, acetic and boric acids 0.04 M were prepared in relation to each of them in the following way: in a 2 dm^3^ volumetric flask, 5.49 cm^3^ H_3_PO_4_ was mixed with 4.58 cm^3^ CH_3_COOH, 4.95 g H_3_BO_3_ was added, the volume was adjusted to 2 dm^3^ with distilled water. A 0.2 M NaOH solution with a volume of 700 cm^3^ was prepared separately. Then, to obtain a buffer solution at the required pH, x cm^3^ of a 0.2 M NaOH solution was added to 100 cm^3^ of the acid solution. The values of the NaOH volumes used with the corresponding pH values are given in Table 3.

### 2.5. Investigation of the Effect of Ionic Force on the Stability of Samples of Se NPs

To study the effect of ionic strength on the stability of positive and negative sols of Se NPs, salt solutions of various concentrations were added to the samples in accordance with Table 4.

At the next stage, samples with a solution of salt and Se NPs were mixed in a volume ratio of 1:1.

## 3. Results and Discussion

In the first stage of the research, the methods of synthesis of Se NPs stabilized with alkyldimethylbenzylammonium chloride (catamine AB) were optimized. Table 5 shows the numerical values of the measured output parameters.

Analysis of the results of optimization of the synthesis technique of Se NPs stabilized with catamine AB showed that the smallest particle size is observed in samples 4, 8, and 9. In turn, a day later, in the samples of colloidal solutions of Se NPs under the numbers 1, 2, 3, 4, 5, 7, and 9, there was a loss of aggregative stability, which was accompanied by coagulation of particles. Thus, aggregative stable samples are No. 6 and 8, with electrokinetic potential values of +9.61 and −2.25, respectively.

In order to determine the optimal conditions for the synthesis of Se NPs stabilized with catamine AB, planning and mathematical processing of the experimental results were carried out in the Neural Statistica Network application software package (Statsoft, Tulsa, OK, USA). The particles size and ζ-potential were chosen as criteria for the stability for Se NPs sols. The concentrations of selenious acid, catamine AB and ascorbic acid were the input parameters that had the greatest influence on the size and ζ-potential of the particles. A neural network was formed in the Neural Statistica Network application package [35].

**Figure 2:**
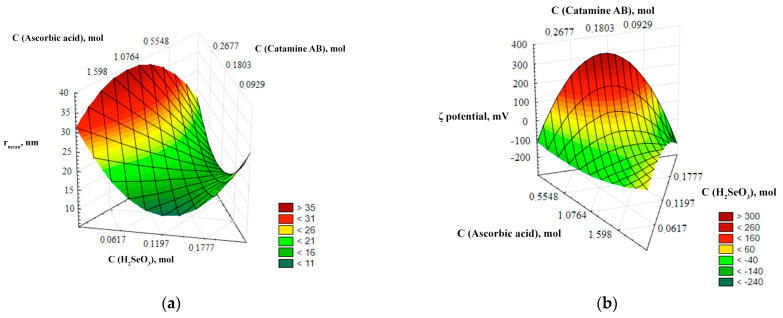
Ternary surfaces describing the relationship with the average hydrodynamic radius (**a**) and ζ-potential (**b**) of Se NPs.

For a more visual representation of the dependencies of the output parameters (r, ζ-potential) on the input parameters (C (catamine), C(H_2_SeO_3_), C(C_6_H_8_O_6_)), three-dimensional ternary dependencies were used. These dependences represent a three-dimensional image describing the relationship of the input parameters with one or more variables under study—the average hydrodynamic radius and the ζ-potential of Se NPs. The ternary dependencies are represented as triangular coordinate systems in space, or on a plane. The ternary surfaces describing the relationship with the average hydrodynamic radius and the ζ-potential of Se NPs are shown in Figure 2.

The analysis of the ternary surfaces of Figure 2 showed that the concentrations of selenious and ascorbic acids have the greatest influence on the average hydrodynamic radius. The minimum size of Se NPs is achieved at a concentration of selenious acid up to 0.17 mol/L and at a concentration of ascorbic acid up to 0.025 mol/L, and the maximum particle size is formed at a concentration of selenious acid about 0.05 mol/L, and at a concentration of ascorbic acid about 1.6 mol/L. The concentration of catamine AB hardly affects the particle size. It is established that the ζ-potential of Se NPs depends significantly on the concentration of acids. At high concentration of ascorbic acid and low concentration of selenious acid, the surface of nanoparticles acquires a negative charge (ζ-potential < 0), and at high concentration of selenious acid and a low concentration of ascorbic acid, the surface of nanoparticles acquires a positive charge (ζ-potential > 0).

**Figure 3:**
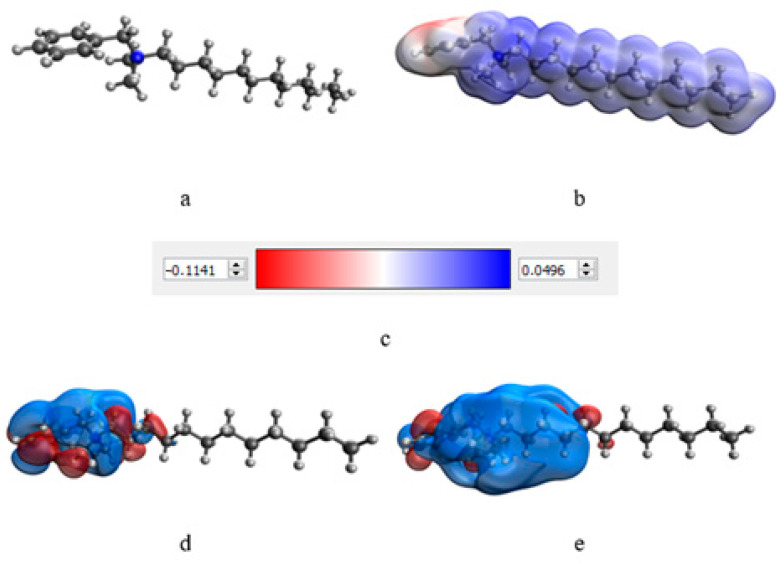
Results of modeling of the catamine AB molecule: molecular complex model (**a**), electron density distribution (**b**), electron density distribution gradient (**c**), highest populated molecular orbital HOMO (**d**), and lowest free molecular orbital LUMO (**e**).

**Figure 4:**
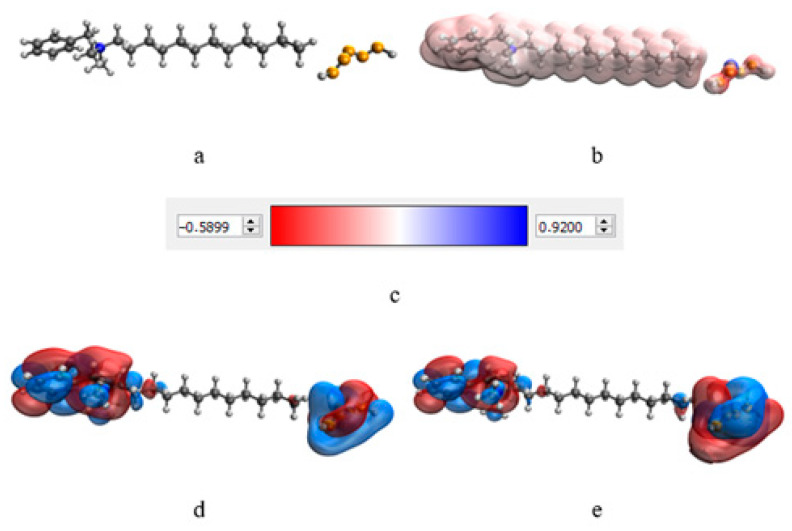
Simulation results of Se NPs stabilized with catamine AB: molecular complex model (**a**), electron density distribution (**b**), electron density distribution gradient (**c**), highest populated molecular orbital HOMO (**d**), lowest free molecular orbital LUMO (**e**).

At the next stage, a quantum-chemical computer simulation of the interaction of the catamine AB molecule with the surface of Se NPs was carried out [36,37]. The data obtained are presented in Figure 3 and Figure 4, and in Table 6.

**Figure 5:**
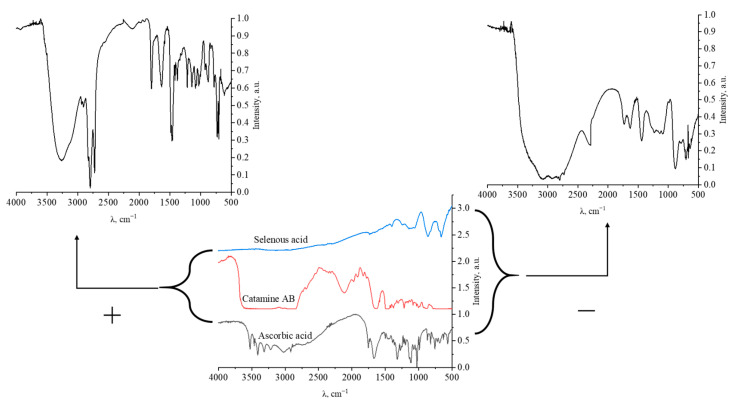
IR spectra of samples of positively and negatively charged Se NP, selenous acid, catamine AB, and ascorbic acid.

The result of quantum-chemical computer modeling revealed that the total energy of the catamine AB molecule was −877.734 kcal/mol, and the energy of the molecular system Se—Catamine AB was −12,869.482 kcal/mol. This fact indicates the energetic benefit of the process of formation of a chemical bond between selenium and catamine AB. In the next stage, the selenium nanoparticles samples were studied by IR Fourier spectroscopy. The resulting spectra are shown in Figure 5.

Table 7 shows the interpretation of the IR spectra of samples of positively and negatively charged NPs of selenium, selenous acid, catamine AB, and ascorbic acid.

The data obtained indicate that in the IR spectrum of Catamine AB, there is a significant decrease in the band in the region from 1580 to 1630 cm^–1^, which is characteristic of the bond radiation of the ionized amino group NH_2_^+^. In the IR spectrum of ascorbic allergy, there is also a decrease in concentration by 1539 cm^−1^, which is characteristic of the ionized carboxyl group COO^-^. Thus, it can be seen that the interaction of surfactant molecules with selenium particles occurs when Se is bound to the charged loads of Catamine AB. It should be noted that in contrast to the positive nanoselenium sol, in the IR spectrum of the negative sol, at 777 cm^−1^, an increased band is observed, characteristic of the vibration of the Se-O bond. This is due to the formation of a potential-forming layer of nanoselenium micelles by the residues of the residue of acids—SeO_3_^2-^, thereby forming the most statement of a negative charge.

**Figure 6:**
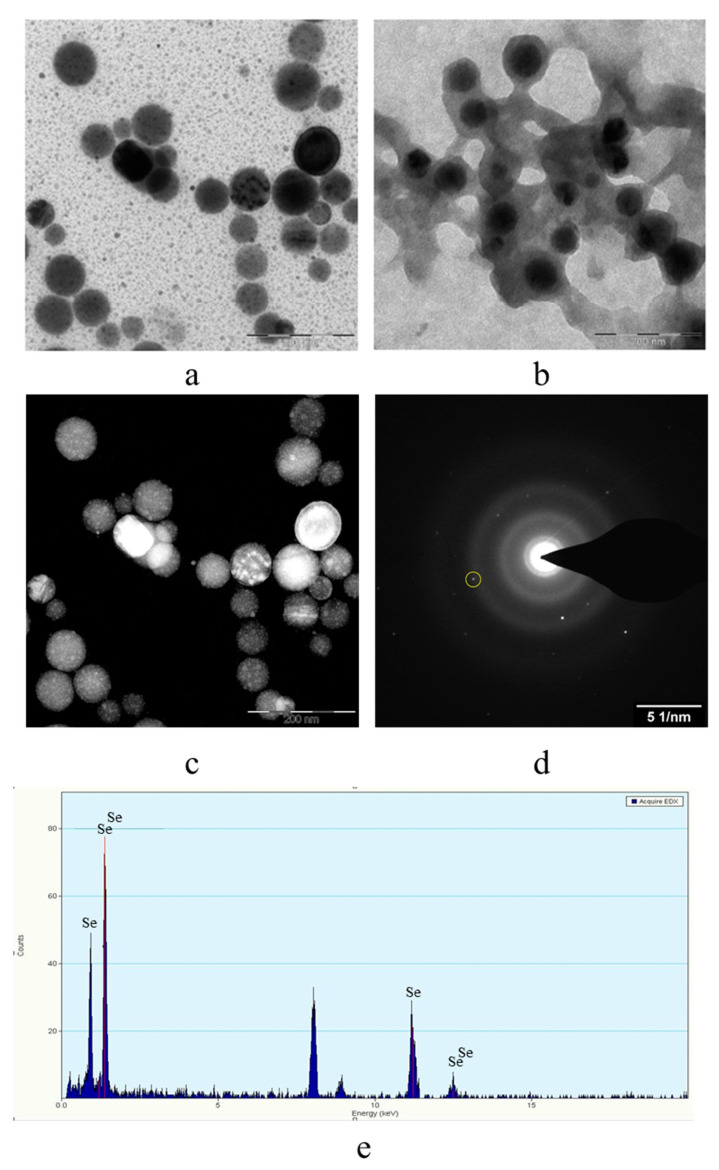
TEM image of samples of Se NPs stabilized with catamine AB: negatively charged sample (**a**), positively charged sample (**b**), dark-field image of one of the particles at higher magnification (**c**); electron diffraction pattern from the selected area (**d**), and spectrum by elements from the selected particle (**e**).

The obtained samples were also examined by transmission electron microscopy on a transmission electron microscope Carl Zeiss Libra 120M. The resulting TEM images are shown in Figure 6.

**Figure 7:**
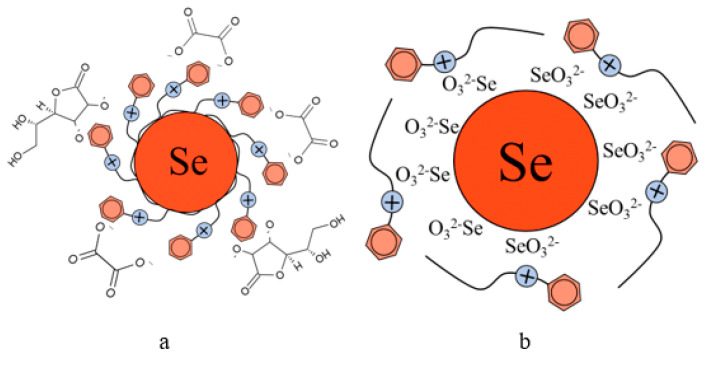
Diagrams of the structure of micelles in samples of Se NPs stabilized with catamine AB: negatively charged sample (**a**), and positively charged sample (**b**).

The analysis of TEM images showed that two fractions of particles are present in the negatively charged samples. The first fraction (smaller in content) is due to the presence of spherical Se NPs with a diameter of 45 ± 10 nm. The second fraction is the largest in content and has a diameter from 1 to 10 nm. The nature of this fraction is associated with the formation of catamine AB micelles that do not contain Se NPs inside. In positively charged samples, one fraction of Se NPs stabilized with catamine AB with a diameter of about 70 nm is observed. It is noteworthy that when alkyldimethylbenzylammonium particles are stabilized with chloride, a thick layer of H is formed on the surface of the Se NPs with a value from 20 to 40 nm. SAED analysis of a sample of negatively charged Se NPs also showed that a single phase of selenium is formed as a result of synthesis. The spectrum obtained as a result of elemental analysis (Figure 6e) also shows Se bands, it is worth noting that there are two copper bands at 8 and 9 keV. These bands correspond to the substrate material on which the Se NPs sample was deposited. Thus, based on the analysis of TEM microphotographs, the proposed schemes of the structure of micelles of positive and negative sols of Se NPs were compiled (Figure 7).

**Figure 8:**
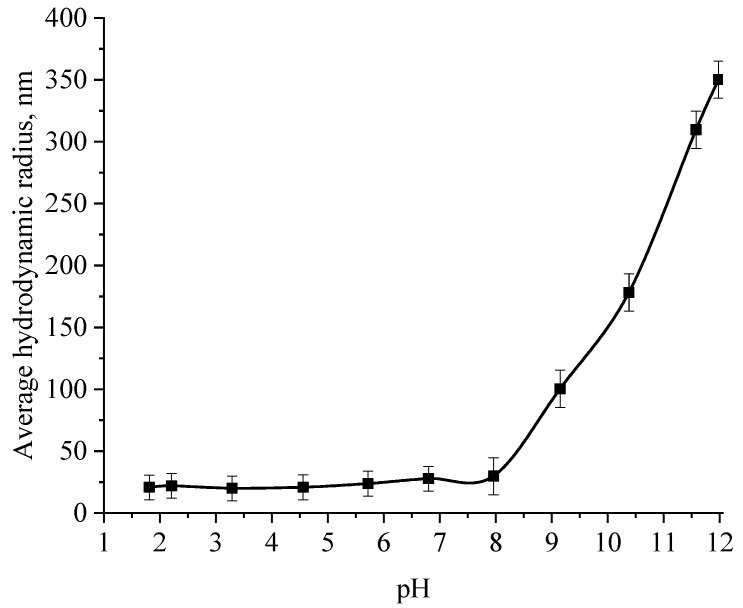
Graph of the dependence of the average hydrodynamic radius of the negative Se NPs sol on the active acidity pH.

**Figure 9:**
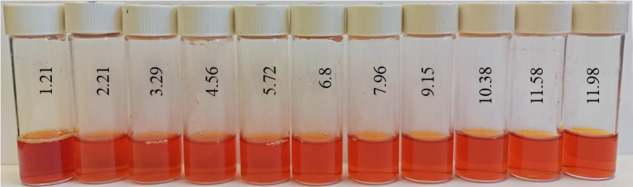
Photo of samples of Se NPs negative sol in various pH media.

**Figure 10:**
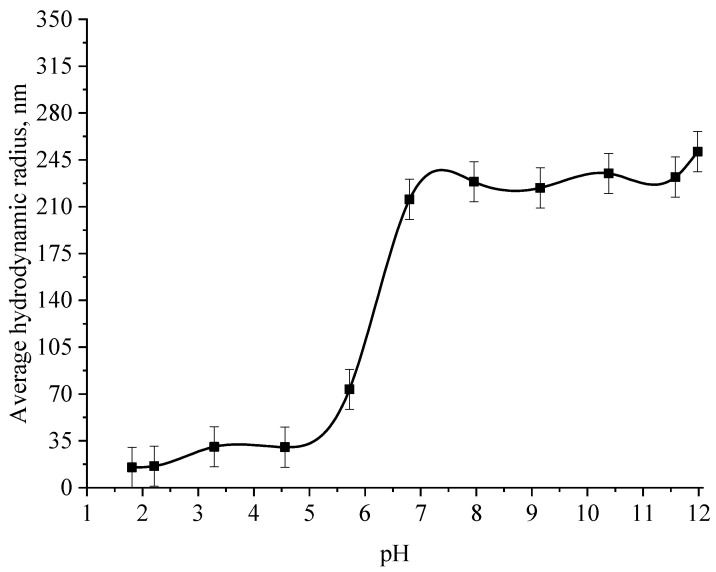
Graph of the dependence of the average hydrodynamic radius of the positive Se NPs sol on the active acidity pH.

**Figure 11:**
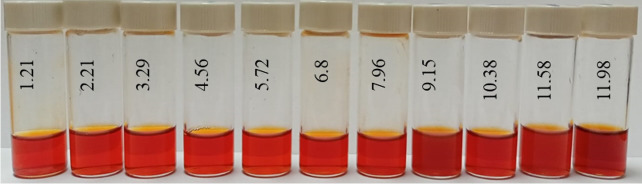
Photo of samples of Se NPs positive sol in various pH media.

At the next stage, the effect of the active acidity of the medium on the average hydrodynamic radius of samples of Se NPs stabilized with alkyldimethylbenzylammonium chloride (catamine AB) was studied. The results are presented in Figure 8, Figure 9, Figure 10 and Figure 11.

From the graph shown in Figure 8, it can be seen that the dependence of the average hydrodynamic radius of Se NPs on the pH of the medium for a negative Se NPs sol is radically different from the same dependence for a positive Se NPs sol. In the pH range from 2 to 8, the change in micelle radii is not significant. An increase in pH above 8 leads to a sharp increase in the size of the part and their coagulation. According to the proposed model of the micelle of Se NPs in a negative sample (Figure 7b), the catamine AB molecules are located in the counterion layer and the diffusion layer. The charge of the micelle is determined by selenious acid ions. A change in the pH of the medium changes the charge of catamine AB from positive in an acidic medium to negative in an alkaline medium. Upon acquiring a negative charge, the molecules begin to repel negatively charged selenious acid ions and diffuse from the surface of nanoparticles [1,38,39]. From the graph shown in Figure 10, it can be seen that in the pH range from 2 to 6, the average size of nanoparticles does not change significantly. In the pH range above 7, particle enlargement and coagulation are noted, and the maximum of the hydrodynamic radius is observed at pH = 12.

**Figure 12:**
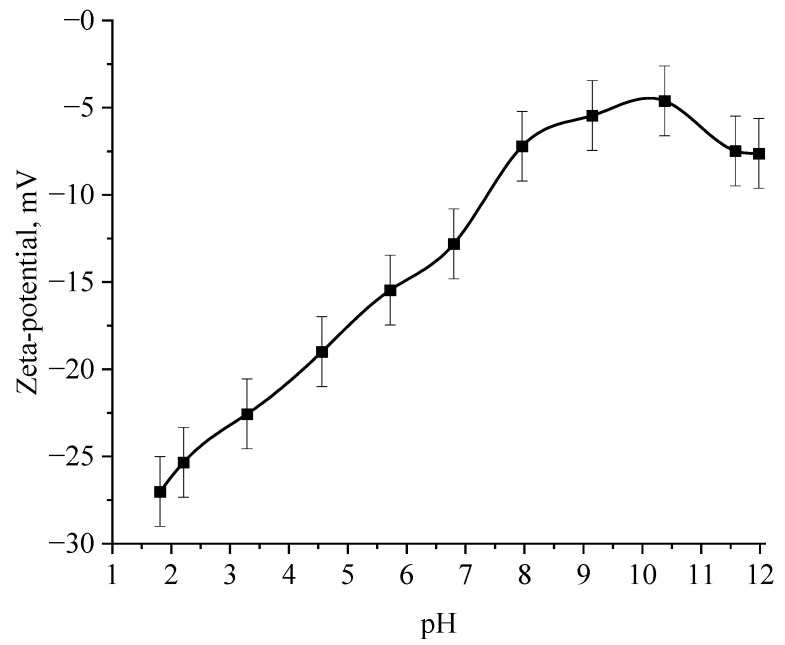
Dependence of the ζ-potential of Se NPs negative sol on the active acidity pH.

**Figure 13:**
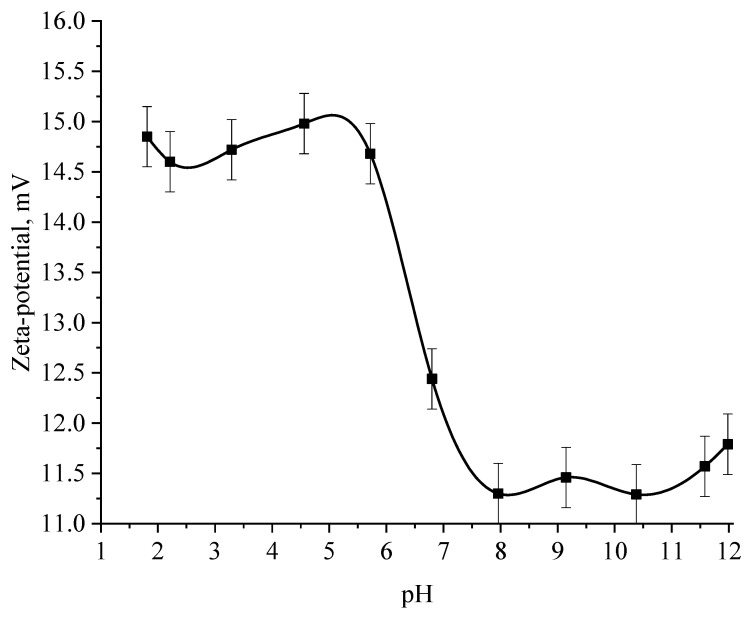
Graph of the dependence of the ζ-potential of Se NPs positive sol on the active acidity pH.

Next, the effect of the active acidity of the medium on the ζ-potential of samples of Se NPs stabilized with alkyldimethylbenzylammonium chloride (catamine AB) was studied. The results are shown in Figure 12 and Figure 13.

The selenious acid adsorbed on the surface of Se NPs is neutralized in alkaline medium, which leads to a loss of stability of the entire system, proven by the graph of the dependence of the ζ-potential of Se NPs negative sol on the active pH acidity.

The analysis of the obtained dependences showed that an increase in the electrokinetic potential is observed in the negative sol with a maximum in the pH range from 11 to 12. It should be noted that an increase in the ζ-potential in the negative sol of the Se NPs is accompanied by an increase in the size and visual coagulation of particles. In turn, the analysis of the dependence of the ζ-potential of Se NPs positive sol on the pH acidity of showed that at pH above 7, there is a decrease in the electrokinetic potential, which leads to an increase in particle size.

**Figure 14:**
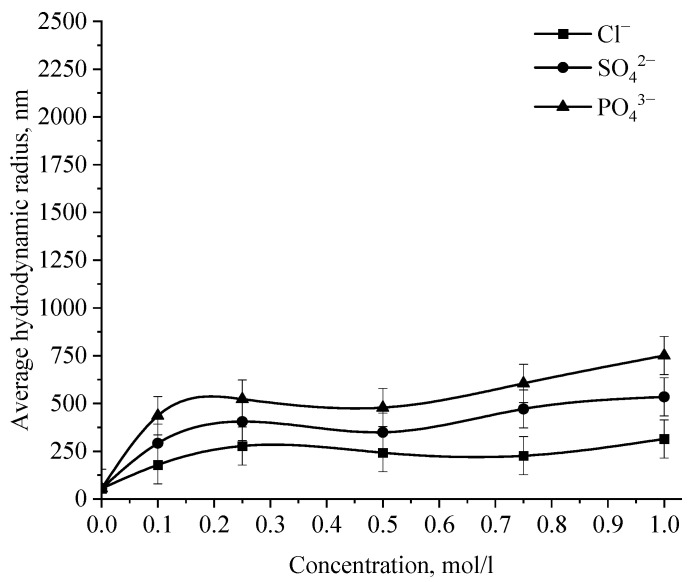
Dependence of the average hydrodynamic radius of Se NPs in negative sol on the concentration of various anions.

**Figure 15:**
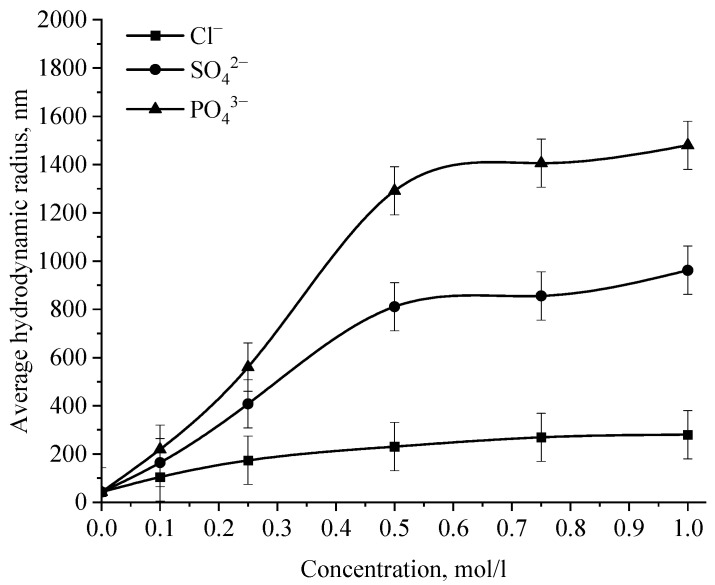
Dependence of the average hydrodynamic radius of Se NPs in positive sol on the concentration of various ions.

**Figure 16:**
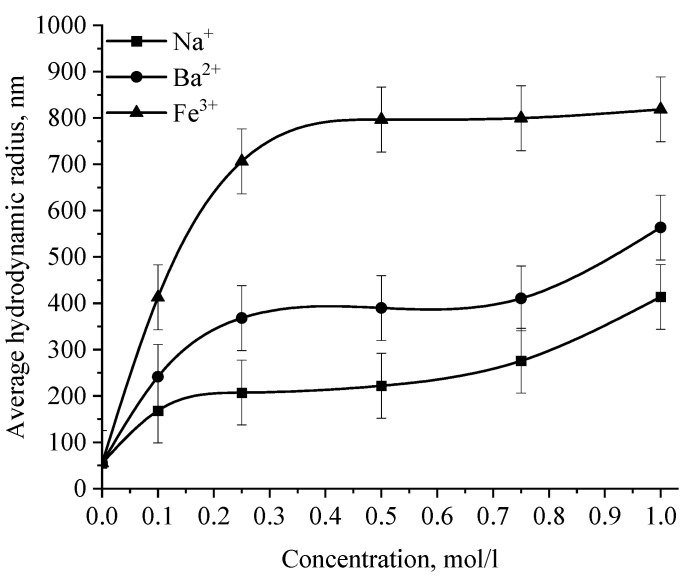
Dependence of the average hydrodynamic radius of Se NPs in negative sol on the concentration of various cations.

**Figure 17:**
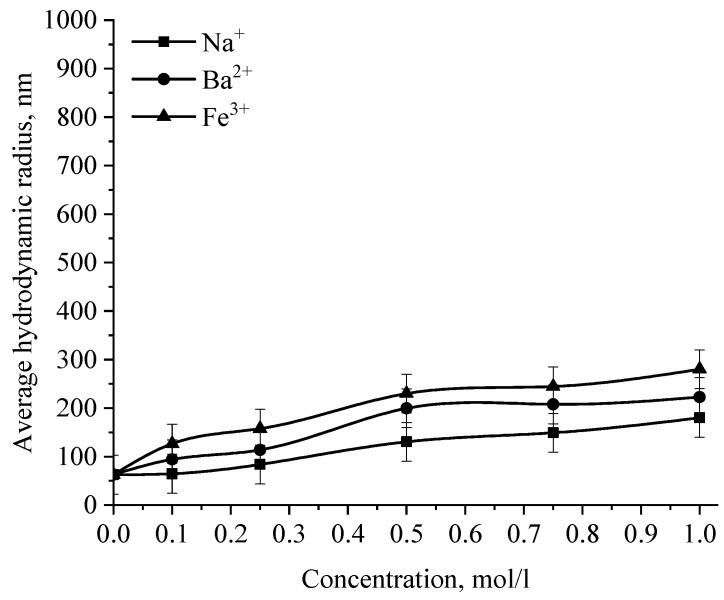
Dependence of the average hydrodynamic radius of Se NPs in positive sol on the concentration of various ions.

In the next stage, the effect of various ions on the stability of the positive and negative sol of Se NPs was investigated. The effect of various cations on the stability of colloidal selenium solutions was analyzed by photon correlation spectroscopy. The results are presented in Figure 14, Figure 15, Figure 16 and Figure 17.

Analysis of the data obtained showed that anions have an insignificant effect on the coagulation of negative sol Se NPs stabilized with alkyldimethylbenzylammonium chloride (catamine AB) [40]. Importantly, the photon correlation spectroscopy results showed that when the salts NaCl, BaCl_2,_ and FeCl_3_ were added to the solution of Se NPs, there was no deviations from the initial results of the average hydrodynamic radius (Figure 15), whereupon further analysis was carried out for Na_2_SO_4_ and Na_3_PO_4_. Figure 15 shows the dependence of the average hydrodynamic radius of positive Se NPs on the concentration of the various ions. 

As shown by the dependencies for phosphate and sulfate ions presented in Figure 15, an increase in the ionic strength of the solution leads to an increase in the size of Se NPs [41,42]. The dependence analysis for the sulfate ion showed that at first glance, an increase in the concentration of the Na_2_SO_4_ solution to 0.3 M does not lead to a change in the radius of the colloidal Se NPs and the solutions remain stable. A further increase in the concentration of the sulfate ion leads to a significant increase in the average hydrodynamic radius of Se NPs. On the following dependence for an electrolyte with a greater coagulating capacity—Na_3_PO_4_, the appearance of turbidity of solutions and precipitation due to the coagulation process of Se NPs is observed the whole concentration considered. At an ion concentration equal to 1 mol/L, the coagulation rate of Se NPs is maximal.

The graph (Figure 16) shows that the increase in the size of colloidal Se NPs occurs with an increase in the concentration of salt in the solution. For Na^+^, there is an increase in the size of colloidal particles from 50 nm to 390 nm, which is not accompanied by a visual change in sol. For Ba^2+^ ions, we can note a sharp increase in the size of negative sol Se NPs from 50 nm to 550 nm at a concentration of Ba^2+^ ions from 0 mol/L to 0.75 mol/L, which is complemented by a visible change in the color of the solution, turning cloudy at an ion concentration of 0.75 mol/L. For the salt with the greatest coagulating effect of Fe^3+^ at the lowest concentration of ions equal to 0.1 mol/L, a visual change in the solution is already noted—the appearance of sediment in the solution and a change in color, and at higher concentrations—a sharp increase in particles up to 800 nm. On the other hand, cations have a negligible effect on the coagulation of positive sol Se NPs stabilized with alkyldimethylbenzylammonium chloride (catamine AB) (Figure 17).

## 4. Conclusions

Thus, in this work, a method was developed for the synthesis of Se NPs stabilized with alkyldimethylbenzylammonium chloride. At the first stage of the study, the synthesis method of Se NPs was optimized using a multifactorial experiment. The analysis of the obtained data showed that the most stable samples are those with a radius of 23.15 and 17.32 nm. Then, the samples were studied by transmission electron microscopy, whose data analysis showed that a bimodal distribution is observed in the negatively charged particles, where one fraction is represented by spheres with a diameter of 45 nm, and the second, from 1 to 10 nm. on the other hand, the positive Se NPs have a diameter of about 70 nm. We also carried out computer quantum-chemical simulations of the stabilization of Se NPs by catamine AB. The analysis of the data obtained confirmed that the use of a stabilizer to obtain Se NPs is energetically more favorable, and the value of the Se-catamine AB configuration was −12869.482 kcal/mol. In the next stage, the influence of the active acidity of the medium on the stability of Se NPs was studied. An analysis of the obtained data showed that both sols of Se NPs exhibit aggregative stability in the pH range from 2 to 6, while an increase in pH to an alkaline medium is accompanied by a loss of particle stability. Next, we studied the effect of ionic strength on the aggregative stability of Se NPs sols. It has been established that negatively charged ions have a significant effect on the particle size of the positive sol of Se NPs, while the particle size of the negative sol is affected by positively charged ions.

The article presents the results of the synthesis of Se NPs stabilized by a quaternary ammonium compound—catamine AB. The synthesis of Se NPs is based on chemical reduction in aqueous medium and is characterized by its simplicity of execution and high reproducibility. Thus, obtained Se NPs stabilized with catamine AB can be used in the perfumery and cosmetics industry, for example, in the production of creams, lotions, shampoos, and other body products with an antioxidant effect. In the future, it is necessary to study the antioxidant properties of such forms of Se NPs, and it is also necessary to carry out series of experiments on laboratory animals.

## Figures and Tables

**Table 1:** Levels of variable variation.

Substance	Parameter Designation	Variable Variation Levels (mg/cm^3^)		
*C* (*H*_2_*SeO*_3_), mg/cm^3^	*a*	0.48	3.8	30.4
*C* (catamine AB), mg/cm^3^	*b*	0.65	5.2	41.6
*C* (ascorbic acid), mg/cm^3^	*c*	5.83	46.6	372.8

**Table 2:** Experiment planning matrix.

Experiment No.	*a* (mg/cm^3^)	*b* (mg/cm^3^)	*c* (mg/cm^3^)
1	0.475	0.65	5.83
2	0.475	5.2	46.6
3	0.475	46.6	372.8
4	3.8	0.65	46.6
5	3.8	5.2	372.8
6	3.8	46.6	5.83
7	30.4	0.65	372.8
8	30.4	5.2	5.83
9	30.4	46.6	46.6

**Table 3:** NaOH volume values with corresponding pH values.

NaOH x cm^3^	pH	NaOH x cm^3^	pH
0	1.81	60	7.96
10	2.21	70	9.15
20	3.29	80	10.38
30	4.56	90	11.58
40	5.72	100	11.98
50	6.8		

**Table 4:** Substances used to study the effect of ionic strength on the stability of samples of Se NPs.

Names of Substances	Concentration of the Substance				
	0.1 M	0.25 M	0.5 M	0.75 M	1 M
	Weight, g				
*NaCl*	0.029	0.073	0.146	0.219	0.29
*Na*_2_ *SO*_4_	0.071	0.178	0.355	0.53	0.71
*K*_3_ *PO*_4_	0.082	0.205	0.41	0.615	0.82
*FeCl*_3_	0.08	0.203	0.406	0.609	0.812
*BaCl*_2_	0.103	0.257	0.514	0.77	1.03

**Table 5:** Results of optimization of the method of synthesis of Se NPs stabilized with catamine AB.

Experiment No.	Parameters	
	r, nm	ζ-Potential, mV
1	20.42	+32.02
2	25.16	+16.5
3	209.81	−62.44
4	18.41	+40.34
5	36.78	+11.59
6	23.15	+9.61
7	25.02	+13.72
8	17.32	−2.25
9	19.79	+1.92

**Table 6:** Results of computer quantum-chemical modeling.

Model	E, kcal/mol	HOMO, eV	LUMO, eV	η, eV
Catamine AB	−877.734	−0.010	0.048	0.029
Catamine AB + Se	−12,869.482	−0.032	−0.027	0.003
Se	−11,991.801	−0.141	−0.042	0.050

**Table 7:** Interpretation of the IR spectra of the obtained samples.

Selenous Acid		Catamine AB		Ascorbic Acid		Positive Sol of Se NPs		Negative Sol of Se NPs	
Band, cm^−1^	Bond	Band, cm^−1^	Bond	Band, cm^−1^	Bond	Band, cm^−1^	Bond	Band, cm^−1^	Bond
417	Se	875	CH_3_	450	CH_2_	659	Se	668	Se
467	Se	997	CH_3_	530	CH_2_	708	Se	701	Se
542	HSeO_3_^−^	1032	C-O	565	CH_3_	729	Se	777	Se-O
569	HSeO_3_^−^	1082	C-O	627	CH_3_	781	CH_3_	882	CH_3_
664	Se-O	1126	C=O	680	C-O	831	CH_3_	982	CH_3_
858	Se-O	1159	C=O	756	C-O	876	CH_3_	1090	C-O
1402	O–H	1203	C=O	868	C-O	926	CH_3_	1136	C=O
1470	O–H	1219	C=O	988	CH_2_	1003	CH_3_	1142	C=O
-	-	1306	CH_3_	1028	C-O	1030	C-O	1236	C=O
-	-	1377	O–H	1076	C-O	1069	C-O	1441	O–H
-	-	1439	O–H	1115	C=O	1082	C=O	1630	NH_2_^+^
-	-	1630	NH_2_^+^	1198	C=O	1140	C=O	1726	C=O
-	-	1780	C=O	1230	C=O	1217	C=O	-	-
-	-	1830	CH_2_	1317	O–H	1379	O–H	-	-
-	-	-	-	1391	O–H	1468	O–H	-	-
-	-	-	-	1499	C-C	1483	C-C	-	-
-	-	-	-	1539	COO^−^	1586	NH_2_^+^	-	-
-	-	-	-	1674	C-C	1634	NH_2_^+^	-	-
-	-	-	-	1753	C=O	1802	C=O	-	-

## Data Availability

All data are available upon request from corresponding author.

## References

[1] Huang Y., Su E., Ren J., Qu X. (2021). The Recent Biological Applications of Selenium-Based Nanomaterials. Nano Today.

[2] Sun H., Jiao R., An G., Xu H., Wang D. (2021). Influence of Particle Size on the Aggregation Behavior of Nanoparticles: Role of Structural Hydration Layer. J. Environ. Sci..

[3] Barvinchenko V.N., Lipkovskaya N.A. (2018). The Effect of a Cationic Gemini Surfactant, Ethonium, on the Physicochemical Properties of Quercetin in Solutions and on the Surface of Highly Dispersed Silica. Colloid J..

[4] Picca R.A., Sportelli M.C., Lopetuso R., Cioffi N. (2017). Electrosynthesis of ZnO Nanomaterials in Aqueous Medium with CTAB Cationic Stabilizer. J. Sol.-Gel. Sci. Technol..

[5] Riedesel S., Kaur R., Bakshi M.S. (2021). Distinguishing Nanoparticle–Nanoparticle Interactions between Gold and Silver Nanoparticles Controlled by Gemini Surfactants: Stability of Nanocolloids. J. Phys. Chem. C.

[6] Juhász Á., Seres L., Varga N., Ungor D., Wojnicki M., Csapó E. (2021). Detailed Calorimetric Analysis of Mixed Micelle Formation from Aqueous Binary Surfactants for Design of Nanoscale Drug Carriers. Nanomaterials.

[7] Xu D., Yang L., Wang Y., Wang G., Rensing C., Zheng S. (2018). Proteins Enriched in Charged Amino Acids Control the Formation and Stabilization of Selenium Nanoparticles in Comamonas Testosteroni S44. Sci. Rep..

[8] Bai Y., Wang Y., Zhou Y., Li W., Zheng W. (2008). Modification and Modulation of Saccharides on Elemental Selenium Nanoparticles in Liquid Phase. Mater. Lett..

[9] Hu Y., Yang H., Wang R., Duan M. (2021). Fabricating Ag@MOF-5 Nanoplates by the Template of MOF-5 and Evaluating Its Antibacterial Activity. Colloids Surf. A Physicochem. Eng. Asp..

[10] Shanmugham V., Subban R. (2022). Capsanthin from Capsicum Annum Fruits Exerts Anti-glaucoma, Antioxidant, Anti-inflammatory Activity, and Corneal Pro-inflammatory Cytokine Gene Expression in a Benzalkonium Chloride-induced Rat Dry Eye Model. J. Food Biochem..

[11] Hedengran A., Begun X., Müllertz O., Mouhammad Z., Vohra R., Bair J., Dartt D.A., Cvenkel B., Heegaard S., Petrovski G. (2021). Benzalkonium Chloride-Preserved Anti-Glaucomatous Eye Drops and Their Effect on Human Conjunctival Goblet Cells in Vitro. Biomed. Hub..

[12] de Carvalho Bernardo W.L., Boriollo M.F.G., Tonon C.C., da Silva J.J., Cruz F.M., Martins A.L., Höfling J.F., Spolidorio D.M.P. (2021). Antimicrobial Effects of Silver Nanoparticles and Extracts of Syzygium Cumini Flowers and Seeds: Periodontal, Cariogenic and Opportunistic Pathogens. Arch. Oral Biol..

[13] Romero G.B., Keck C.M., Müller R.H., Bou-Chacra N.A. (2016). Development of Cationic Nanocrystals for Ocular Delivery. Eur. J. Pharm. Biopharm..

[14] Bashir M., Ali S., Farrukh M.A. (2020). Green Synthesis of Fe_2_O_3_ Nanoparticles from Orange Peel Extract and a Study of Its Antibacterial Activity. J. Korean Phys. Soc..

[15] Caglayan M.G., Kasap E., Cetin D., Suludere Z., Tamer U. (2017). Fabrication of SERS Active Gold Nanorods Using Benzalkonium Chloride, and Their Application to an Immunoassay for Potato Virus X. Microchim. Acta.

[16] Abdul-Moqueet M.M., Tovias L., Lopez P., Mayer K.M. (2021). Synthesis and Bioconjugation of Alkanethiol-Stabilized Gold Bipyramid Nanoparticles. Nanotechnology.

[17] Ansari S.A., Alshanberi A.M. (2021). Clinical Application of Silver Nanoparticles Coated by Benzalkonium Chloride. Coatings.

[18] Brumovský M., Micić V., Oborná J., Filip J., Hofmann T., Tunega D. (2023). Iron Nitride Nanoparticles for Rapid Dechlorination of Mixed Chlorinated Ethene Contamination. J. Hazard. Mater..

[19] Dement’eva O.V., Frolova L.V., Rudoy V.M., Kuznetsov Y.I. (2016). Sol–Gel Synthesis of Silica Containers Using a Corrosion Inhibitor, Catamine AB, as a Templating Agent. Colloid J..

[20] Ozcelikay G., Dogan-Topal B., Ozkan S.A. (2018). An Electrochemical Sensor Based on Silver Nanoparticles-Benzalkonium Chloride for the Voltammetric Determination of Antiviral Drug Tenofovir. Electroanalysis.

[21] Bjørklund G., Shanaida M., Lysiuk R., Antonyak H., Klishch I., Shanaida V., Peana M. (2022). Selenium: An Antioxidant with a Critical Role in Anti-Aging. Molecules.

[22] Ozturk Kurt B., Ozdemir S. (2022). Selenium in Food Chain in Relation to Human and Animal Nutrition and Health.

[23] Reich H.J., Hondal R.J. (2016). Why Nature Chose Selenium. ACS Chem. Biol..

[24] Garza-García J.J.O., Hernández-Díaz J.A., Zamudio-Ojeda A., León-Morales J.M., Guerrero-Guzmán A., Sánchez-Chiprés D.R., López-Velázquez J.C., García-Morales S. (2022). The Role of Selenium Nanoparticles in Agriculture and Food Technology. Biol. Trace Elem. Res..

[25] Ferro C., Florindo H.F., Santos H.A. (2021). Selenium Nanoparticles for Biomedical Applications: From Development and Characterization to Therapeutics. Adv. Healthc. Mater..

[26] Alfthan G., Eurola M., Ekholm P., Venäläinen E.-R., Root T., Korkalainen K., Hartikainen H., Salminen P., Hietaniemi V., Aspila P. (2015). Effects of Nationwide Addition of Selenium to Fertilizers on Foods, and Animal and Human Health in Finland: From Deficiency to Optimal Selenium Status of the Population. J. Trace Elem. Med. Biol..

[27] Blinov A.V., Kostenko K.V., Gvozdenko A.A., Maglakelidze D.G., Golik A.B., Nagdalian A.A., Statsenko E.N., Nikulnikova N.N., Remizov D.M., Verevkina M.N. (2021). Study of Stabilization of Selenium Nanoparticles by Polysaccharides. J. Hyg. Eng. Des..

[28] Shurygina I.A., Shurygin M.G. (2020). Use of Nanoselenium in Chemotherapy Drug Delivery Systems. Nanotechnol. Russ..

[29] Hu T., Liang Y., Zhao G., Wu W., Li H., Guo Y. (2019). Selenium Biofortification and Antioxidant Activity in Cordyceps Militaris Supplied with Selenate, Selenite, or Selenomethionine. Biol. Trace Elem. Res..

[30] Kumar A., Prasad K.S. (2021). Role of Nano-Selenium in Health and Environment. J. Biotechnol..

[31] Hosnedlova B., Kepinska M., Skalickova S., Fernandez C., Ruttkay-Nedecky B., Peng Q., Baron M., Melcova M., Opatrilova R., Zidkova J. (2018). Nano-Selenium and Its Nanomedicine Applications: A Critical Review. Int. J. Nanomed..

[32] Kalinin S.V., Ophus C., Voyles P.M., Erni R., Kepaptsoglou D., Grillo V., Lupini A.R., Oxley M.P., Schwenker E., Chan M.K.Y. (2022). Machine Learning in Scanning Transmission Electron Microscopy. Nat. Rev. Methods Prim..

[33] Lee J., He S., Song G., Hogan C.J. (2022). Size Distribution Monitoring for Chemical Mechanical Polishing Slurries: An Intercomparison of Electron Microscopy, Dynamic Light Scattering, and Differential Mobility Analysis. Powder Technol..

[34] Mohammadi-Jam S., Waters K.E., Greenwood R.W. (2022). A Review of Zeta Potential Measurements Using Electroacoustics. Adv. Colloid Interface Sci..

[35] Kattenborn T., Leitloff J., Schiefer F., Hinz S. (2021). Review on Convolutional Neural Networks (CNN) in Vegetation Remote Sensing. ISPRS J. Photogramm. Remote Sens..

[36] Salim E., Ali Hassan R.R. (2022). Alkyl Dimethyl Benzyl Ammonium Chloride as a New Cleaner for Washing Treatments for Historical Printed Paper. Pigment. Resin Technol..

[37] Siddiqui S.A., Blinov A.V., Serov A.V., Gvozdenko A.A., Kravtsov A.A., Nagdalian A.A., Raffa V.V., Maglakelidze D.G., Blinova A.A., Kobina A.V. (2021). Effect of Selenium Nanoparticles on Germination of Hordéum Vulgáre Barley Seeds. Coatings.

[38] Guleria A., Baby C.M., Tomy A., Maurya D.K., Neogy S., Debnath A.K., Adhikari S. (2021). Size Tuning, Phase Stabilization, and Anticancer Efficacy of Amorphous Selenium Nanoparticles: Effect of Ion-Pair Interaction, −OH Functionalization, and Reuse of RTILs as Host Matrix. J. Phys. Chem. C.

[39] Song X., Chen Y., Sun H., Liu X., Leng X. (2021). Physicochemical Stability and Functional Properties of Selenium Nanoparticles Stabilized by Chitosan, Carrageenan, and Gum Arabic. Carbohydr. Polym..

[40] Gao X., Ren K., Zhu Z., Zhang J., Li S., Wang J., Xu Y. (2023). Specific Ion Effects: The Role of Anions in the Aggregation of Permanently Charged Clay Mineral Particles. J. Soils Sediments.

[41] Pal P., Malhotra M. (2022). Emerging Technologies for Selenium Separation and Recovery from Aqueous Systems: A Review for Sustainable Management Strategy. Can. J. Chem. Eng..

[42] Li C., Hassan A., Palmai M., Snee P., Baveye P.C., Darnault C.J.G. (2022). Colloidal Stability and Aggregation Kinetics of Nanocrystal CdSe/ZnS Quantum Dots in Aqueous Systems: Effects of Ionic Strength, Electrolyte Type, and Natural Organic Matter. SN Appl. Sci..

